# A method for hand-foot-mouth disease prediction using GeoDetector and LSTM model in Guangxi, China

**DOI:** 10.1038/s41598-019-54495-2

**Published:** 2019-11-29

**Authors:** Jiangyan Gu, Lizhong Liang, Hongquan Song, Yunfeng Kong, Rui Ma, Yane Hou, Jinyu Zhao, Junjie Liu, Nan He, Yang Zhang

**Affiliations:** 1 Laboratory of Geospatial Technology for the Middle and Lower Yellow River Regions, Ministry of Education, Henan University, Kaifeng, Henan 475004 China; 2 Institute of Urban Big Data, College of Environment and Planning, Henan University, Kaifeng, Henan 475004 China; 3 The Affiliated Hospital of Guangdong Medical University, Zhanjiang, 524001 China; 4 Henan Key Laboratory of Integrated Air Pollution Control and Ecological Security, Henan University, Kaifeng, Henan 475004 China; 5 Institute for Global Innovation and Development, East China Normal University, Shanghai, 200062 China

**Keywords:** Viral infection, Risk factors

## Abstract

Hand-foot-mouth disease (HFMD) is a common infectious disease in children and is particularly severe in Guangxi, China. Meteorological conditions are known to play a pivotal role in the HFMD. Previous studies have reported numerous models to predict the incidence of HFMD. In this study, we proposed a new method for the HFMD prediction using GeoDetector and a Long Short-Term Memory neural network (LSTM). The daily meteorological factors and HFMD records in Guangxi during 2014–2015 were adopted. First, potential risk factors for the occurrence of HFMD were identified based on the GeoDetector. Then, region-specific prediction models were developed in 14 administrative regions of Guangxi, China using an optimized three-layer LSTM model. Prediction results (the R-square ranges from 0.39 to 0.71) showed that the model proposed in this study had a good performance in HFMD predictions. This model could provide support for the prevention and control of HFMD. Moreover, this model could also be extended to the time series prediction of other infectious diseases.

## Introduction

Hand-foot-mouth disease (HFMD) is a common viral infectious disease in children under 5 years old, which is commonly caused by the enteric pathogen coxsackievirus A16 (CoxA16) and enterovirus 71 (EV 71)^1,2^. Severe HFMD could be associated with serious complications, such as poliomyelitis and brainstem encephalitis, which may be life-threatening^3^. HFMD has resulted in several outbreaks throughout the world and become a public health issue in Asia^4–7^. HFMD ranked first among the notifiable infectious diseases in China in 2017^8^. The incidence and mortality rate of HFMD are particularly severe in Guangxi Zhuang Autonomous Region of China^9^. There are no specific drugs or vaccines to prevent HFMD^10,11^ and therefore it is essential to establish a reliable prediction model for the prevention of HFMD.

HFMD has obvious periodic variations, such as its peak period usually occurs during summer months in the northern hemisphere^12^. Previous studies have revealed that HFMD is closely related to meteorological conditions^13–16^, such as the mean temperature, relative humidity, wind speed, and sunshine hours. It should be possible to establish models to predict the occurrence of HFMD based on these associations. The prediction model would enable individuals as well as hospitals and clinics formulate precautions and minimize health risks.

Numerous studies have been carried out to develop HFMD prediction models. There are three categories of prediction models, including linear regression, time series, and machine learning. The linear regression model was established by analyzing the correlations between the incidence of HFMD and the influential factors^17^. However, it is difficult to capture the non-linear association between HFMD and impacting factors and maintain the spatial stationary assumption over a large area^18^. The time series model uses the relationship in the sequential lag time series to predict the incidence of HFMD, such as the seasonal auto-regressive integrated moving average model (ARIMA)^19,20^. These models did not consider the relationship between HFMD and potential impacting factors. With the development of artificial intelligence (AI), machine learning algorithms have shown their advantages in predictions and recognitions^21–23^. Gradient boosting tree (GBT) and random forest (RF) were found to be capable of identifying both mild and severe HFMD, which is helpful for early surveillance and control in HFMD^24,25^. Deep learning methods such as Back Propagation Neural Networks (BPNN) were also adopted to predict the incidence of HFMD^26^. However, conventional machine learning methods such as BPNN cannot effectively deal with the trend prediction of HFMD since the temporal pattern must be taken into account when predicting infectious diseases.

To overcome the limitations mentioned above, this study proposed a HFMD prediction method using the GeoDetector (http://geodetector.org) and the Long Short-Term Memory Neural Network (LSTM). GeoDetector measures the association between input factors and dependent factors according to their temporal-spatial distributions by the indicator *q*-statistic (*q*) value, the value ∈ [0,1] increases as the association between the input factor and HFMD increase^27^. LSTM is an advanced kind of Recurrent Neural Network (RNN), which has the ability to learn temporal pattern and store the useful memory for a longer time. In this study, the GeoDetector was employed to analyze the impact of every meteorological factor and the interactive effects between different factors on HFMD. And then the dominant impacting factors were input into LSTM model to predict the weekly cases of HFMD in 14 subregions of Guangxi, China.

## Results

### Identification of potential impacting factors

**Figure 1:**
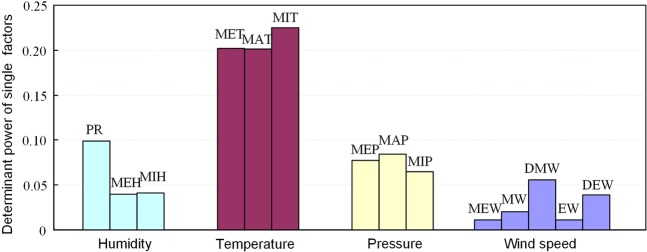
Determinant power of the potential impacting factors of HFMD.

Figure 1 shows the contributions of 14 meteorological factors to the occurrence of HFMD in 14 subregions of Guangxi from 2014 to 2015. The 14 meteorological factors were divided into four categories, including (1) Humidity: minimum relative humidity (MIH), mean relative humidity (MEH), and precipitation (PR); (2) temperature: mean temperature (MET), maximum temperature (MAT), and minimum temperature (MIT); (3) pressure: mean pressure (MEP), maximum pressure (MAP), and minimum pressure (MIP); (4) wind speed: mean wind speed (MEW), maximum wind speed(MW), the direction of maximum wind speed (DMW), extreme wind speed (EW), and the direction of extreme wind speed (DEW). It can be seen that the primary impacting factor is the temperature of MIT (*q* = 0.23) and MET (*q* = 0.20), followed by PR (*q* = 0.10) and wind speed (DEW, *q* = 0.04; MW, *q* = 0.02; EW, *q* = 0.01; MEW, *q* = 0.01). This indicated that the *q* value was similar for the category of potential impacting factors, which means that they may have the similar contribution to the occurrence of HFMD, but does not mean that they influence the HFMD in the same way.

**Figure 2:**
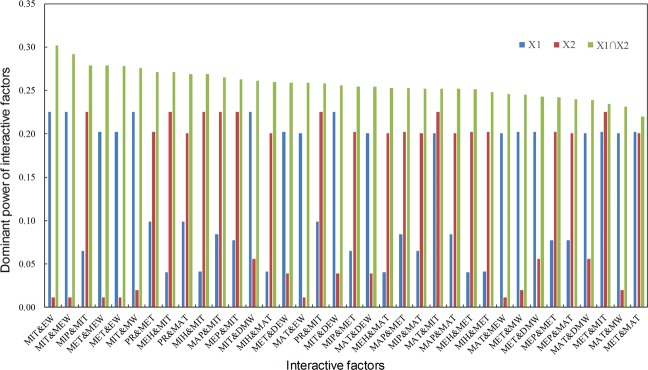
Interactive effects between the potential influencing factors on HFMD. The x-axis label *X*1 & X2 denotes q values of *X*1 (blue), *X*2 (red), and the interaction between *X*1 and *X*2 (green).

The interactive effect is not simply equal to the sum of the *q* values of the two influencing factors’ (*X*1 and *X*2) effects on HFMD, which is represented as *q*(*X*1 ∩ *X*2)^28^. Figure 2 shows the interactive results between potential influencing factors (only show a subset (*q* (*X*1 ∩ *X*2) > 0.20) of all the interactions due to the space limitation, entire results could be found in the Supplementary Fig. S1). This indicated that any two combined factors could play a more important role than their single effects on HFMD. The combinations of the temperatures and other factors had more dominated influences among all the combinations. The most four primary interactions MIT and EW (*q* = 0.30), MIT and MEW (*q* = 0.30), MIT and MIP (*q* = 0.28), and MET and MEW (*q* = 0.28). Taking the effects of both single and combined effects into consideration, MW and DEW were eliminated from the risk factors.

### LSTM prediction model

To minimize the spatial difference of the effects of meteorological conditions on HFMD and improve the prediction accuracy of LSTM, we developed the region-specific models for 14 subregions of Guangxi. The HFMD cases of the previous 80 weeks were taken as the training set to train the LSTM model, and the HFMD cases of the next 24 weeks were taken as the testing set to evaluate the prediction model. The model was saved after 5000 iterations, which was applied to predict the HFMD cases in the subsequent 24 weeks. We run 20 times for each model and the mean value of the runs was considered as the prediction value.

**Figure 3:**
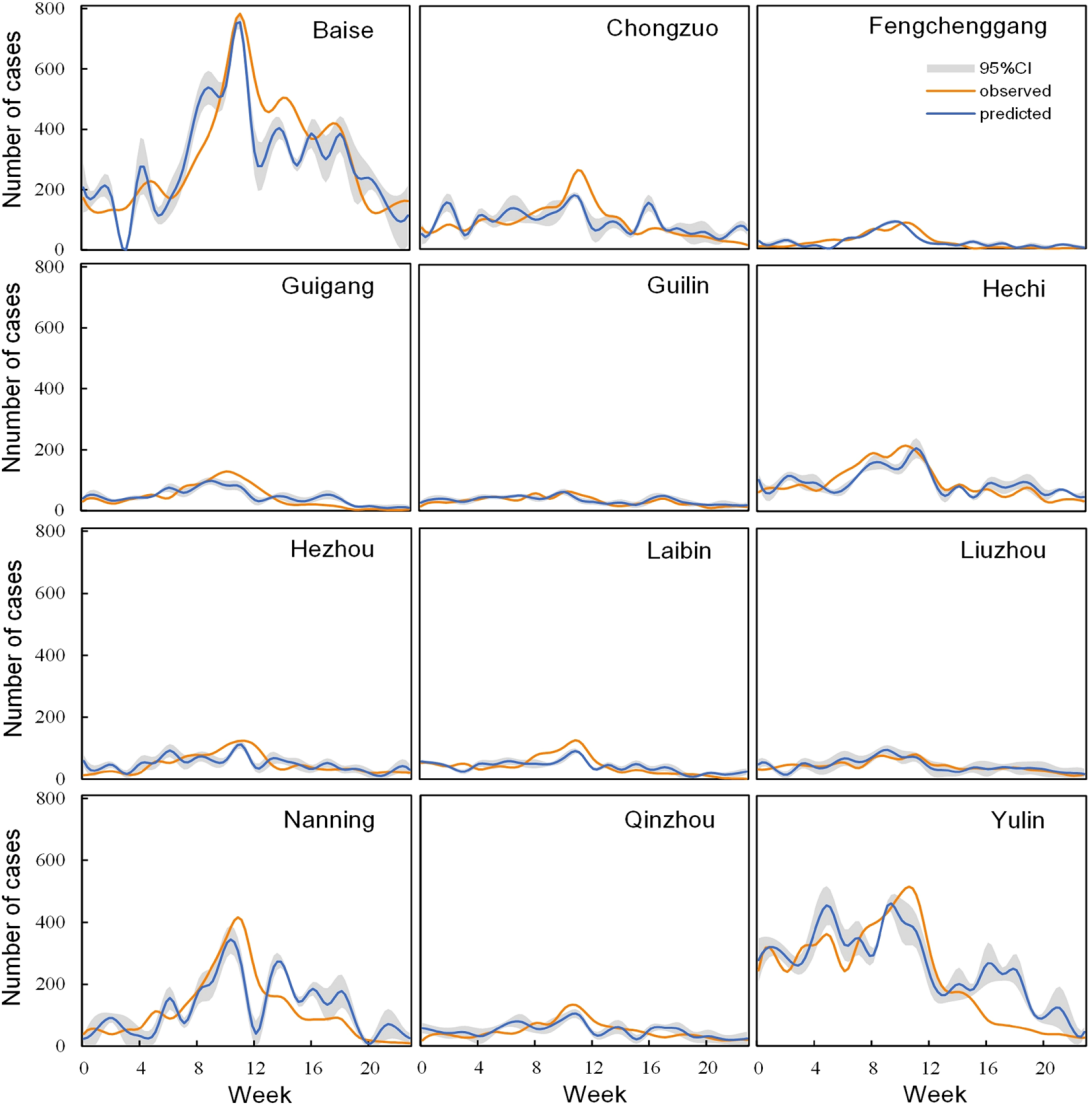
Region-specific model predictions of HFMD compared with observations in subregions. The grey shaded areas demote the 95% confidence interval (CI) of the predictions.

Figure 3 shows predictions of the region-specific LSTM models compared with observations in subregions (Beihai and Wuzhou not shown here due to the lower cases of HFMD, entire results could be found in the Supplementary Fig. S2). The predictions and observations had good consistence in all subregions, which indicated that the region-specific LSTM models had good performance in the prediction of HFMD. To quantify the performance of the region-specific models, the metrics of R Square (*R*^2^) and Mean Absolute Percent Error (*MAPE*) were adopted to evaluate the performance of these models. Table 1 shows the performance of the 14 region-specific models in Guangxi. The mean *R*^2^ and *MAPE* of these models was 0.60 and 0.73, respectively. The *R*^2^ ranges from 0.39 to 0.71 and the *MAPE* ranges from 23% to 131%. The region-specific model showed best and worst performance in Baise (*R*^2^ = 0.71, *MAPE* = 30.46%) and Chongzuo (*R*^2^ = 0.39, *MAPE* = 55.29%), respectively.

**Table 1:** The performance of the region-specific models in subregions of Guangxi.

Region	Chongzuo	Hezhou	Qinzhou	Liuzhou	Nanning	Beihai	Guilin
R^2^	0.39	0.40	0.49	0.54	0.56	0.60	0.61
MAPE	0.55	0.52	0.43	0.23	0.92	1.32	0.35
**Region**	**Laibin**	**Wuzhou**	**Guigang**	**Yulin**	**Fangchenggang**	**Hechi**	**Baise**
R^2^	0.64	0.65	0.68	0.68	0.70	0.70	0.71
MAPE	1.05	1.38	0.96	0.75	1.07	0.36	0.30

## Discussion

It has been reported that the incidence of HFMD significantly increased in recent years in China^29^, particularly in the southeast areas. This study proposed a method for predictions of the HFMD occurrence using GeoDetector and LSTM model.

Primary potential impacting factors for HFMD were identified by the GeoDetector. Compared with the conventional regression models, the GeoDetector can not only identify non-linear associations but also detect interactive effects from multiple variables. According to the detection results of the GeoDetector, the *q* values of the temperatures and the precipitation are at high levels, indicating that they could be crucial influential factors of the HFMD. Particularly, the temperatures rank higher associations than other variables. In addition, interactions of temperatures and wind speeds rank highest, which means that these combinations play an important role in the occurrence of HFDM. This finding is consistent with previous studies^13–16^. Existing researches explained the association between climate and disease, such as wind can promote circulation and distribution of air pollutants like particulate matter carrying enterovirus to accelerate the transportation of HFMD^30^. Temperature change could not only influence the children’s immune capacity^31^, but can also influence human’s direct contact to increase the opportunity of HFMD transmissions, as physical activities among individuals are increasing in warm months^32^. A study in Hefei, China, has found that more than half of HFMD cases occurred on rainy days, because the wet weather is suitable for the HFMD virus to survive and multiply^33^.

Predictions of the region-specific models in 14 subregions of Guangxi proved that LSTM has the ability to predict the occurrence of HFMD. Numerous studies have contributed to the construction of HFMD prediction model^18–20^. However, this may be the first time to adopt the LSTM, to the best of our knowledge, to predict the occurrence of HFMD based on meteorological conditions. The mean *R*^2^ and MAPE of the 14 region-specific models was 0.60 and 0.73, respectively. Compared with previous works predicted by other methods^34,35^ (mean *R*^2^ = 0.54, mean MAPE = 1.02), indicating that the model proposed in this study had higher accuracy in the HFMD predictions. However, the performance of the LSTM model had spatial variations, which may be due to the meteorological conditions may not be the primary driving factors in some regions. For example, the socio-economic factors may be the primary driving forces for the incidence of HFMD^36^.

It should also be noted that there were some limitations in the region-specific LSTM prediction model proposed in this study. The training dataset may be partial and insufficient due to the uneven distribution of hospitals, which may result in uncertainties for the prediction of HFMD. However, we believe that this can capture the temporal trends of HFMD occurrence for each subregion, because the dataset adopted in this study was collected continuously from most hospitals in each region. In addition, we adopted normalized and optimized method to minimize the prediction errors in the model construction. Moreover, the HFMD is also affected by socio-economic factors such as population density, rural population, and proportion of student population^17,36^. However, we only considered meteorological factors in this study, which may possibly lead to inaccurate predictions in regions where meteorological factors are not strong determinants of the HFMD. Taking other potential impacting factors into the LSTM model development would improve the prediction accuracy of HFMD occurrence.

In conclusion, this study proposed a method for predicting HFMD using GeoDetector and LSTM. The method was proved to be accurate and effective. Although this model cannot be applied directly in other studies due to the parameters in deep learning algorithm models vary with training data, the framework proposed in this study can be extended to predict other infectious diseases in other study areas. The capability of LSTM in dealing with time series issues could be applied more extensively in further researches.

## Materials and Methods

### Data sources

**Figure 4:**
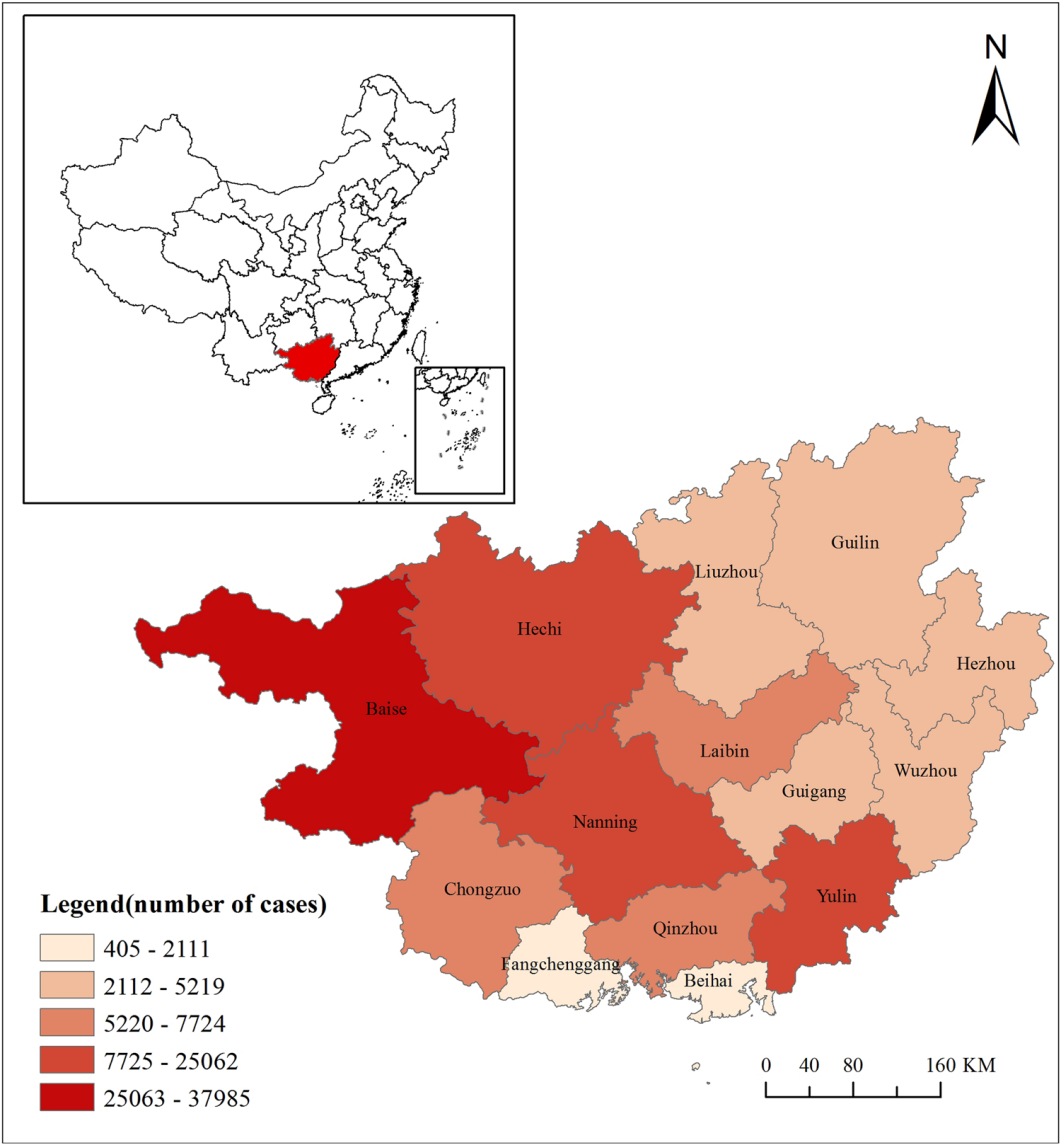
Location of Guangxi Zhuang Autonomous Region in China and the total number of HFMD cases during 2014–2015.

We collected the daily historical first page data of medical records and meteorological data from January 2014 to December 2015 in Guangxi, China. Guangxi is located in southeast China, adjacent to the South China Sea. Most of it is in the subtropical zone with a monsoon humid and rainy climate (Fig. 4).

The first page data of medical records for hospitals were collected from 14 administrative regions of Guangxi, including patients age, source, admission time, hospital stay, diagnosis, operation, payment, and the form of payment. Considering the scale of the first page data, the big data technologies such as data cleaning and denoising were adopted during the first page data preprocessing. The HFMD cases were defined according to the International Classification of Diseases B08.4. Finally, there were 170920 records of HFMD were adopted in this study, which were divided by 14 administrative regions and 104 weeks.

Meteorological data were obtained from the China Meteorological Data Sharing Service System. The original data were collected at 99 meteorological stations in and around Guangxi, including 14 meteorological factors, MIH, MEH, PR, MET, MAT, MIT, MEP, MAP, MIP, MEW, MW, DMW, EW, and DEW. In order to get the meteorological data for each week and each region, a model equipped with iterator was built by using the Model Builder function of ArcMap 10 (https://desktop.arcgis.com/en/arcmap/). The main function of this model was to turn the daily station records to weekly region records by spatiotemporal Kriging interpolation method. Spatiotemporal interpolation method was an extended interpolation way based on spatial temporary relativity, considering the variables both in space and time, the estimated value of a time-space spot is calculated by the weighted sum of its surrounding observations. Main methods include spatiotemporal Kriging, BME (Bayesian Maximum Entropy), and synthesis method^37^. Among all the spatial interpolation ways, the spatiotemporal Kriging was a simple way and commonly used for the interpolation of climatic data^38,39^.

### GeoDetector

GeoDetector is a statistical method to detect the temporal-spatial heterogeneity. This tool has been widely used in many areas, such as heavy metal differentiation, land use, and disease risk factor detection^40–42^. The assumption is that, if a potential factor leads to a disease, this factor would show a temporal-spatial distribution similar to the disease. In this study, GeoDetector was adopted to identify the risk factors from the 14 candidate meteorological factors that caused the temporal spatial stratified heterogeneity of HFMD in Guangxi from 2014 to 2015. Then, the impacting factors were identified according to the ranking of *q* values. The calculation of *q* is as follows:

1 $$q=1-\frac{{\sum }_{h=1}^{L}{\sum }_{i=1}^{{N}_{h}}{({Y}_{hi}-{\bar{Y}}_{h})}^{2}}{{\sum }_{i=1}^{N}{({Y}_{i}-\bar{Y})}^{2}}$$

This equation assumes that the study area is composed of *N* units and is stratified into h ∈ [1, 2, 3_……_L] strata, *Y*_*i*_ is the value of sample *i*, *i* is the whole sample population, ${Y}_{hi}$ means the value of sample *i* in stratum *h*, ${\bar{Y}}_{h}$ is the mean value of stratum *h*, $\bar{Y}$ is the mean value of population. A higher value of *q* indicates a stronger spatially stratified heterogeneity of *Y*; it means that factor *X* can explain 100 * *q* % of the temporal-spatial pattern of *Y*. Moreover, according to the rules and principle of GeoDetector, the *X* should be a categorized variable instead of numerical variables^28,43^, therefore, the continuous meteorological factors were categorized into six levels using *k*-means cluster algorithm.

### LSTM neural networks

LSTM is a special kind of RNN (Recurrent Neural Network). RNN has the ability to learn patterns and extract features from data that contains time series due to the special structure in context layer, a hidden layer with repeated connections in neurons. LSTM has been widely used in recognitions of image and speech due to its high accuracy^44,45^. However, it remains a long-term dependency issue in RNN due to the exploding gradient problem resulting from gradient propagation over many layers. LSTM was designed to overcome this issue through cell-and-gate structure, which enables the LSTM to learn when they forget and update memory^46^. LSTM has a better performance than tradition statistical models and has been applied in predicting such as emotional state, traffic flow, and disease, especially when combined with convolutional neural networks^47–49^.

**Figure 5:**
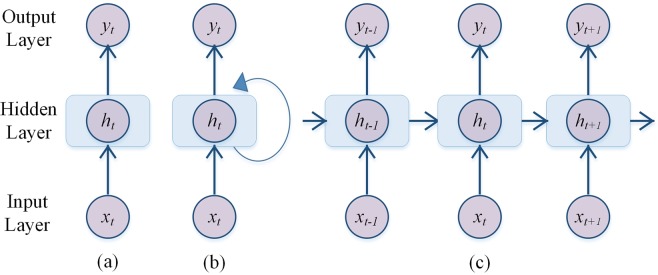
Architecture of artificial neural networks. (**a**) Architecture of feed-forward neural network. (**b**) Architecture of RNN. (**c**) Architecture of RNN unfolded in time.

In order to learn the pattern in time series, the conventional deep feed-forward neural networks must be improved (Fig. 5a). The lack of connections among the nodes within the hidden layers may result in failure in dealing with time series problems. Therefore, RNN, a kind of neural network equipped with recurrent connections in the neurons of hidden layers, has been developed and improved^50^. Figure 5b,c show a basic RNN architecture and unfolded architecture in time. The connections or loops can transport feedback from the previous state to the current state, allowing information to be passed between the consecutive temporal steps. Hence, it can be seen that the output not only depends on the input information, but also depends on the output of the previous hidden layer.

The model in Fig. 5a can be expressed mathematically as follows:

2 $${h}_{t}={f}_{1}({w}_{1}{x}_{t}+{b}_{1})$$

3 $${y}_{t}={f}_{2}({w}_{2}{h}_{t}+{b}_{2})$$

where *x*_*t*_ is the input variable, *h*_*t*_ is the temporary variable in hidden layer or hidden state; *w*_1_ and *w*_2_ are weight metrics from input layer to hidden layer and from hidden layer to output layer, respectively; *b*_1_ and *b*_2_ are bias vectors; *f*_1_, and *f*_2_ are hidden and output activation functions, respectively. The activation functions are nonlinear functions, making the neural networks approximate any continual nonlinear functions with any precision^51^.

The mathematical expressions of the feedback loop in a hidden layer are as follow equations (Fig. 5b):

4 $${h}_{t}={f}_{1}({w}_{t1}{x}_{t}+{w}_{t2}{h}_{t-1}+{b}_{1})$$

5 $${y}_{t}={f}_{2}({w}_{2}{h}_{t}+{b}_{2})$$

where the *w*_*t*1_, *w*_*t*2_ and *w*_2_ are weight matrices; *b*_1_, *b*_2_, *f*_1_ and *f*_2_ have the same meaning as described for conventional deep feed-forward neural networks. The same weights are used at each time step to calculate the output *y*_*t*_.The loop makes the *h*_*t*_ at time *t* calculated not only by the input *x*_*t*_ but also by the previous output *h*_*t*−1_, which is consistent with the unfolded RNN architecture shown in Fig. 5b. It can be seen that the temporal information is continuously reflected over time. The RNN is actually a very deep neural network trained using back propagation algorithm in time direction. However, due to the vanishing gradient or exploding gradient problems that always occur in very deep neural networks^52^, the accuracy of the RNN deteriorates quickly over a long period of time, which means the RNN can only store short-term memory. This is called the long-term dependency of RNNs.

**Figure 6:**
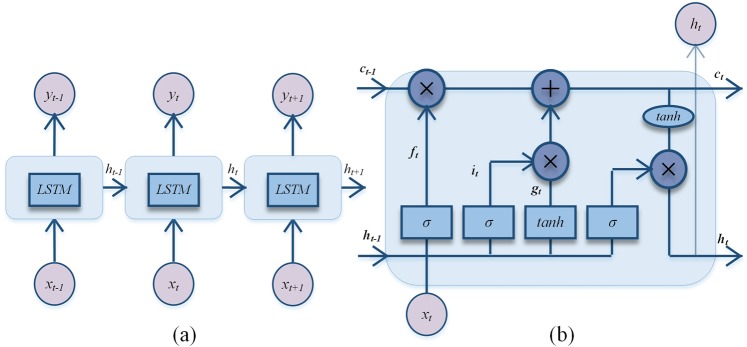
Architecture of LSTM. (**a**) Architecture of LSTM. (**b**) Architecture of LSTM memory unit.

The LSTM was proposed by Sepp Hochreiter and Jürgen Schmidhuber to overcome the long-term dependency of RNNs in 1997^53^. For LSTM, the hidden neurons of the RNN are replaced by LSTM memory units (Fig. 6a). The memory units are mainly composed of three gates and one cell (Fig. 6b), which aim to control information flow and storage, including input gate, forget gate, output gate and memory cell. The system is determined by the state of these structures at each time step whether the information will be retained. Thus, the LSTM could hold important short-term memory for a longer period by the filtration of the memory units.

The formulas for calculations in the memory unit are performed as follows:

6 $${i}_{t}=\sigma ({w}_{i1}{x}_{t}+{w}_{i2}{h}_{t-1}+{b}_{i})$$

7 $${f}_{t}=\sigma ({w}_{f1}{x}_{t}+{w}_{f2}{h}_{t-1}+{b}_{f})$$

8 $${o}_{t}=\sigma ({w}_{o1}{x}_{t}+{w}_{o2}{h}_{t-1}+{b}_{0})$$

9 $${g}_{t}=tanh({w}_{g1}{x}_{t}+{w}_{g2}{h}_{t-1}+{b}_{g})$$

10 $${c}_{t}={f}_{t}\ast {c}_{t-1}+{i}_{t}\ast {g}_{t}$$

11 $${h}_{t}={o}_{t}\ast \,\tanh \,({c}_{t})$$

here, the input gate *i*_*t*_, forget gate *f*_*t*_, and output gate *o*_*t*_ take variable *x*_*t*_ and previous hidden state *h*_*t−*1_ as inputs at time *t*, multiplied with the weight matrices ${w}_{i1}$ ${w}_{i2}\,{w}_{f1}\,{w}_{f2}\,{w}_{o1}{x}_{t}\,{w}_{o2}$, then plus bias vectors *b*_*i*_, *b*_*f*_, *b*_*o*,_ *σ* means the sigmoid function $\sigma (z)={(1+{e}^{-z})}^{-1}$, generating the outputs of these gates range from 0 to 1. The larger output value is, the more information allowed, i.e., if the output value equals 1, it means the information is fully entered. The *c*_*t*−1_ means previous state of memory cell; the * indicates element-wise multiplication. *tanh* is a kind of activation function such that, $tanh(z)=\frac{{e}^{x}-{e}^{-x}}{{e}^{x}+{e}^{-x}}$. The calculations are as follows: after inputting the candidate information *g*_*t*_ to be stored, the actual amount stored is determined by the input gate *i*_*t*_. Then in the Eq.(10), the final cell sta_*t*_e *c*_*t*_ depends on the sum of the amount of newly input informat*i*on *i*_*t*_ * *g*_*t*_ and the amount of forgotten information $\,{f}_{t}\ast {c}_{t-1}$, *h*_*t*_ means the final output of the memory unit, controlled by the output gate *o*_*t*_ and the final cell state *c*_*t*_. The LSTM model was constructed in Python 3.5 and was supported by modules including Tensorflow, Numpy, and Pandas.

### Model design

In order to identify the risk meteorological factors of the temporal-spatial distribution of HFMD in Guangxi, the preprocessed data were sent to GeoDetector, including weekly normalized cases and categorized meteorological factors in each region. In addition, the weekly cases need to be normalized in data preprocessing due to the collected data cannot cover all the hospitals, so the case number instead of incidence was adopted as dependent variable. However, the number of cases between regions had large spatial differences because of the uneven distribution of hospitals and population. To solve this issue, we standardizes the number of cases to eliminate dimension. Z-score was adopted to normalize the data. The Z-score calculation method is:

12 $${x}^{\ast }=\frac{x-\bar{x}}{\sqrt{\frac{1}{N}}{\sum }_{i=1}^{N}{({x}_{i}-\bar{x})}^{2}}$$

The environmental factors are different in different regions of Guangxi. To minimize the effect of spatial scale and improve the accuracy of LSTM, the region-specific model was built for each of the 14 regions of Guangxi. After the identification by using GeoDetector, the identified impacting factors and weekly cases in any given region were used as inputs and outputs, respectively. To develop the LSTM prediction model, the hospital data of the first 80 weeks work as the training set and the last 24 weeks work as the testing set. The number of input layers was equal to the number of identified factors and the neuron in hidden layer was set as 10 after experiments and adjustments. Because the output is a continuous variable, the output layer was set as 1 without activation functions. To study the model with continuous variables, the root-mean-square error was set as loss function. Backward propagation with gradient descent was used as a training algorithm to minimize the result of loss function. The optimization of the LSTM prediction model had three parts: L2 regularization, moving average model and exponential decay learning rate. First, in order to avoid the overfitting problem caused by the limited number of data, L2 regularization was adopted in loss function as shown in equations^54^, where *c*_0_ denotes the original loss function; the ε denotes the regularization rate, usually set as a very small number, such as 0.0001 in this paper; and $\sum _{w}{w}^{2}$ indicates the sum of squares of all the weights.

13 $${c}_{0}=\frac{1}{n}\mathop{\sum }\limits_{i=1}^{n}{({y}_{pi}-{y}_{i})}^{2}$$

14 $${\rm{c}}={c}_{0}+\frac{\varepsilon }{2n}\sum _{w}{w}^{2}$$

Second, the moving average model is always accompanied by a gradient decay algorithm to improve the capacity and extensiveness of the final output model. Shadow variables following the variables are held and updated with iterations by the model to control the update rate^55^, after experiments, the decay rate was determined as 0.99.

15 $$shado{w}_{i}={\rm{decay}}\_{\rm{rate}}\ast shado{w}_{i-1}+(1-{\rm{decay}}\_{\rm{rate}})\ast variabl{e}_{i}$$

The other important parameter in neural networks is learning rate. It has been proved that dynamic rate has a better effect than fixed rate in training^56^. Therefore, in this study, an exponential decay learning rate was adopted, and the mathematical mechanism is expressed in Eq. (16). A better solution is using a higher learning rate at first, called learning rate base, then it will decrease gradually as the increase in global step over time. The learning rate base was determined as 0.1, and the decay rate was 0.99 after experiments.

16 $${\rm{learning}}\_{\rm{rate}}={\rm{base}}\_{\rm{rate}}\ast {(decay\_rate)}^{\frac{global\_step}{decay\_steps}}$$

### Ethics approval

The present study was approved by the medical and research ethics committee of Henan University (see the ethical approval in the related manuscript file), all methods were carried out in accordance with relevant guidelines and regulations. Since there were no individual information involved and the records are anonymized in this study, the approving IRB has waived the need to collect informed consent, so the informed consent was not required in the present study.

## Supplementary information

supplementary figures

Dataset 1

Dataset 2

## Data Availability

The meteorological dataset used during the current study are available from China Meteorological Data Sharing Service System (http://data.cma.cn/). The hospital datasets can be extracted as presented in the website of data collection, or contact the corresponding author on reasonable request.
