# Supplementary material for: A method for hand-foot-mouth disease prediction using GeoDetector and LSTM model in Guangxi, China
Source: Sci Rep. 2019 Nov 29;9:17928. doi: 10.1038/s41598-019-54495-2 (PMC6884467; doi:10.1038/s41598-019-54495-2)
Supplement: Supplementary file 1 — supplementary figures [file 41598_2019_54495_MOESM1_ESM.pdf]

# A method for hand-foot-mouth disease prediction using Geodetector and LSTM model in Guangxi, China

Jiangyan Gu[1, 2], Lizhong Liang[3], Hongquan Song[1, 2, 4*], Yunfeng Kong[1, 2*], Rui Ma[1], Yane Hou[1], Jinyu Zhao[1], Junjie Liu[1], Nan He[1], Yang Zhang[5]

[1] Laboratory of Geospatial Technology for the Middle and Lower Yellow River Regions, Ministry of Education, Henan University, Kaifeng, Henan 475004, China

[2] Institute of Urban Big Data, College of Environment and Planning, Henan University, Kaifeng, Henan 475004, China

[3] The Affiliated Hospital of Guangdong Medical University, Zhanjiang 524001, China

[4] Henan Key Laboratory of Integrated Air Pollution Control and Ecological Security, Henan University, Kaifeng, Henan 475004, China

[5] Institute for Global Innovation and Development, East China Normal University, Shanghai, 200062, China

*Corresponding author: hqsong@henu.edu.cn (H. Song); yfkong@henu.edu.cn (Y. Kong)

Address: College of Environment and Planning, Henan University Jinming Campus, Kaifeng, Henan Province 475004, China

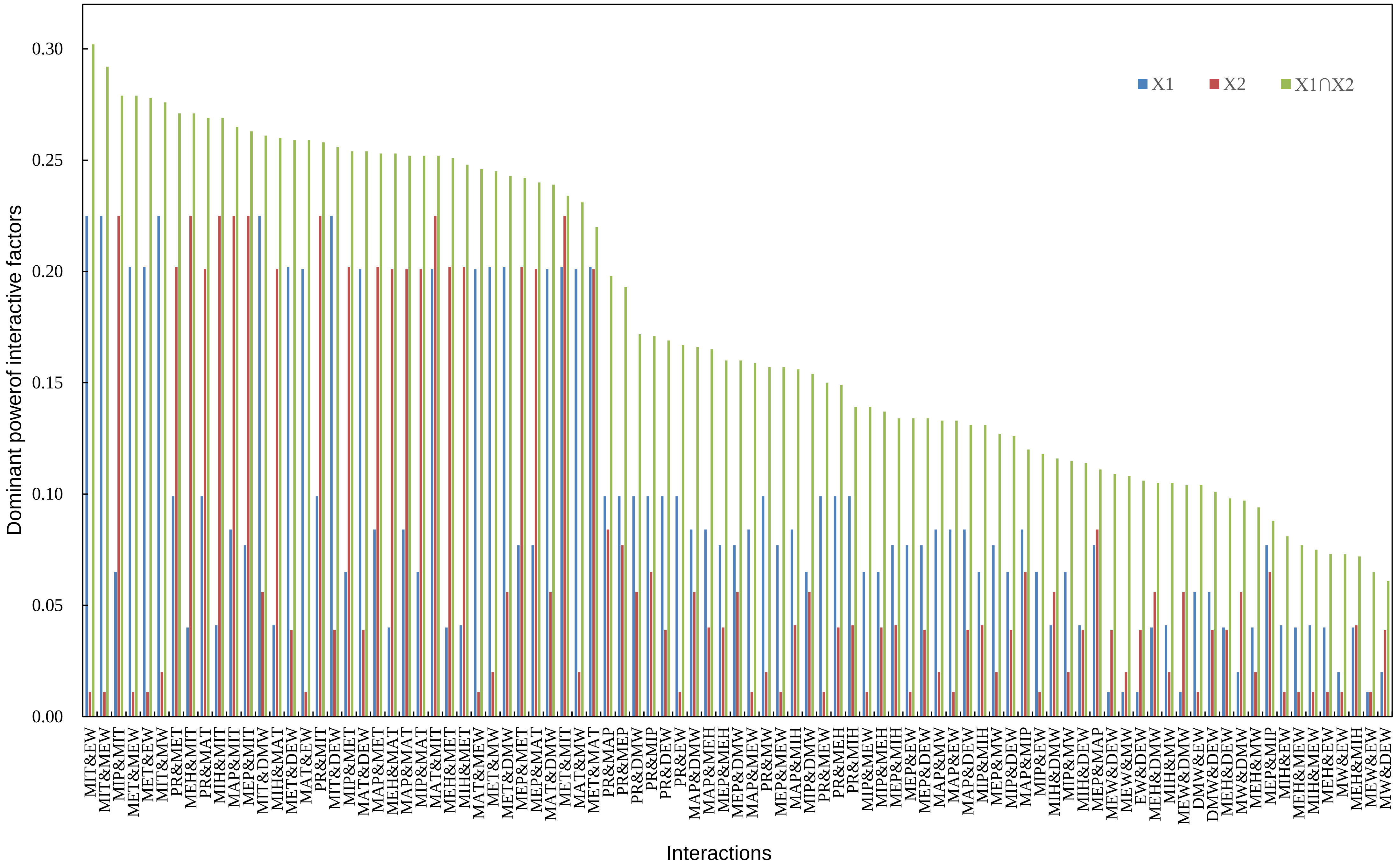


**Figure S1. The entire interactive effects between the potential influencing factors on HFMD.**

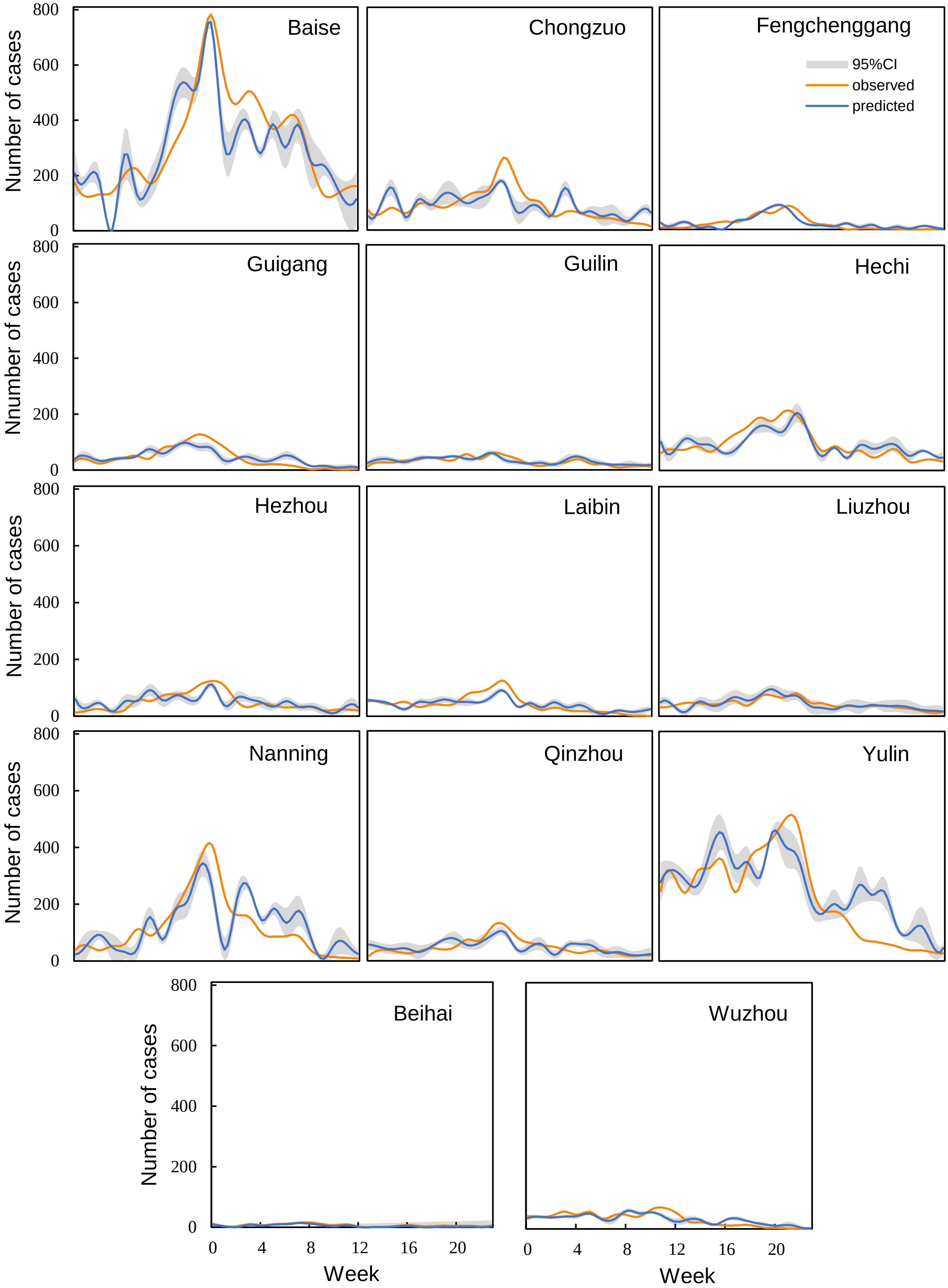


**Figure S2. The entire region-specific model predictions of HFMD compared with observations in subregions. The grey shaded areas demote to the 95% confidence interval of the predictions.**
